# A New Work for the Dental Profession

**Published:** 1854-01

**Authors:** 


### A NEW WORK FOR THE DENTAL PROFESSION.

This is a work on Chemistry and Metallurgy, as applied to the study and practice of Dental Surgery, by Prof. A. Snowden Piggot, M. D. The Professionhas long needed just such a work, and we are glad that one so able has undertaken and accomplished the work.

The work is divided into four distinct parts, or arranged under four separate heads, and a mere glance will show the importance of the subjects treated of.

Under the first head we have a consideration of the ultimate chemical elements of the human body. This part of the work is very full and complete and its importance can only be duly appreciated when we remember that a proper knowledge of these elements forms the proper basis from which we must study the complete organization of the human body.

In the second part we have digestion considered in all its physiological relations. This subject, considered as the great producing power in all the operations of assimilation, is identified in the perfect developement of every organ, and to the Dentist its careful study is of the utmost consequence.

The third division treats of the "Chemistry of the Mouth." Here we have the teeth and the saliva brought under chemical review, giving all the different changes we may expect in the morbid condition of the saliva and secretions from the mucous membrane.

In the fourth part we have "Chemistry and Metallurgy of the metals and earths used by the Dentist." This subject commences with the blow-pipe and goes on through the minute detail of the refining, alloying and working of all the metals used, and closes with a history of the materials used and manner of making incorruptible teeth, giving the latest improvements in this department of dental science. We think the work is one which should be in the hands of every dentist, and although we have not as yet had much time for its perusal, yet we have looked over it enough to see that much labor and research has been spent on it, and an immense amount of useful information combined and given in a very desirable form to the Profession.

